# Neuroprotective effect of curcumin against experimental autoimmune encephalomyelitis-induced cognitive and physical impairments in mice: an insight into the role of the AMPK/SIRT1 pathway

**DOI:** 10.1007/s10787-023-01399-3

**Published:** 2023-12-19

**Authors:** Mohamed A. Sadek, Mostafa A. Rabie, Nesrine S. El Sayed, Helmy M. Sayed, Esraa A. Kandil

**Affiliations:** Department of Pharmacology and Toxicology, Faculty of Pharmacy, Cairo University, Cairo, Egypt

**Keywords:** Multiple sclerosis, EAE, Curcumin, Cognition, AMPK/SIRT1, Nrf2

## Abstract

Multiple sclerosis (MS) is an incurable chronic neurodegenerative disease where autoimmunity, oxidative stress, and neuroinflammation collaboration predispose myelin sheath destruction. Interestingly, curcumin, a natural polyphenol, showed a neuroprotective effect in numerous neurodegenerative diseases, including MS. Nevertheless, the influence of curcumin against MS-induced cognitive impairment is still vague. Hence, we induced experimental autoimmune encephalomyelitis (EAE) in mice using spinal cord homogenate (SCH) and complete Freund’s adjuvant, which eventually mimic MS. This study aimed not only to evaluate curcumin efficacy against EAE-induced cognitive and motor dysfunction, but also to explore a novel mechanism of action, by which curcumin exerts its beneficial effects in MS. Curcumin (200 mg/kg/day) efficacy was evaluated by behavioral tests, histopathological examination, and biochemical tests. Concisely, curcumin amended EAE-induced cognitive and motor impairments, as demonstrated by the behavioral tests and histopathological examination of the hippocampus. Interestingly, curcumin activated the adenosine monophosphate (AMP)-activated protein kinase/silent mating type information regulation 2 homolog 1 (AMPK/SIRT1) axis, which triggered cyclic AMP response element-binding protein/brain-derived neurotrophic factor/myelin basic protein (CREB/BDNF/MBP) pathway, hindering demyelination of the corpus callosum. Furthermore, AMPK/SIRT1 activation augmented nuclear factor erythroid 2-related factor 2 (Nrf2), a powerful antioxidant, amending EAE-induced oxidative stress. Additionally, curcumin abolished EAE-induced neuroinflammation by inhibiting Janus kinase 2 /signal transducers and activators of transcription 3 (JAK2/STAT3) axis, by various pathways, including AMPK/SIRT1 activation. JAK2/STAT3 inhibition halts inflammatory cytokines synthesis. In conclusion, curcumin’s neuroprotective effect in EAE is controlled, at least in part, by AMPK/SIRT1 activation, which ultimately minimizes EAE-induced neuronal demyelination, oxidative stress, and neuroinflammation.

**Graphical Abstract:**

Graphical illustration of putative molecular pathways implicated in the management of EAE by curcumin. Curcumin activates AMPK/SIRT1, which in turn activates multiple pathways that hinder neurodegeneration, oxidative stress, and neuroinflammation. Moreover, curcumin conquers the inflammatory pathway JAK2/STAT3/NF-kβ.

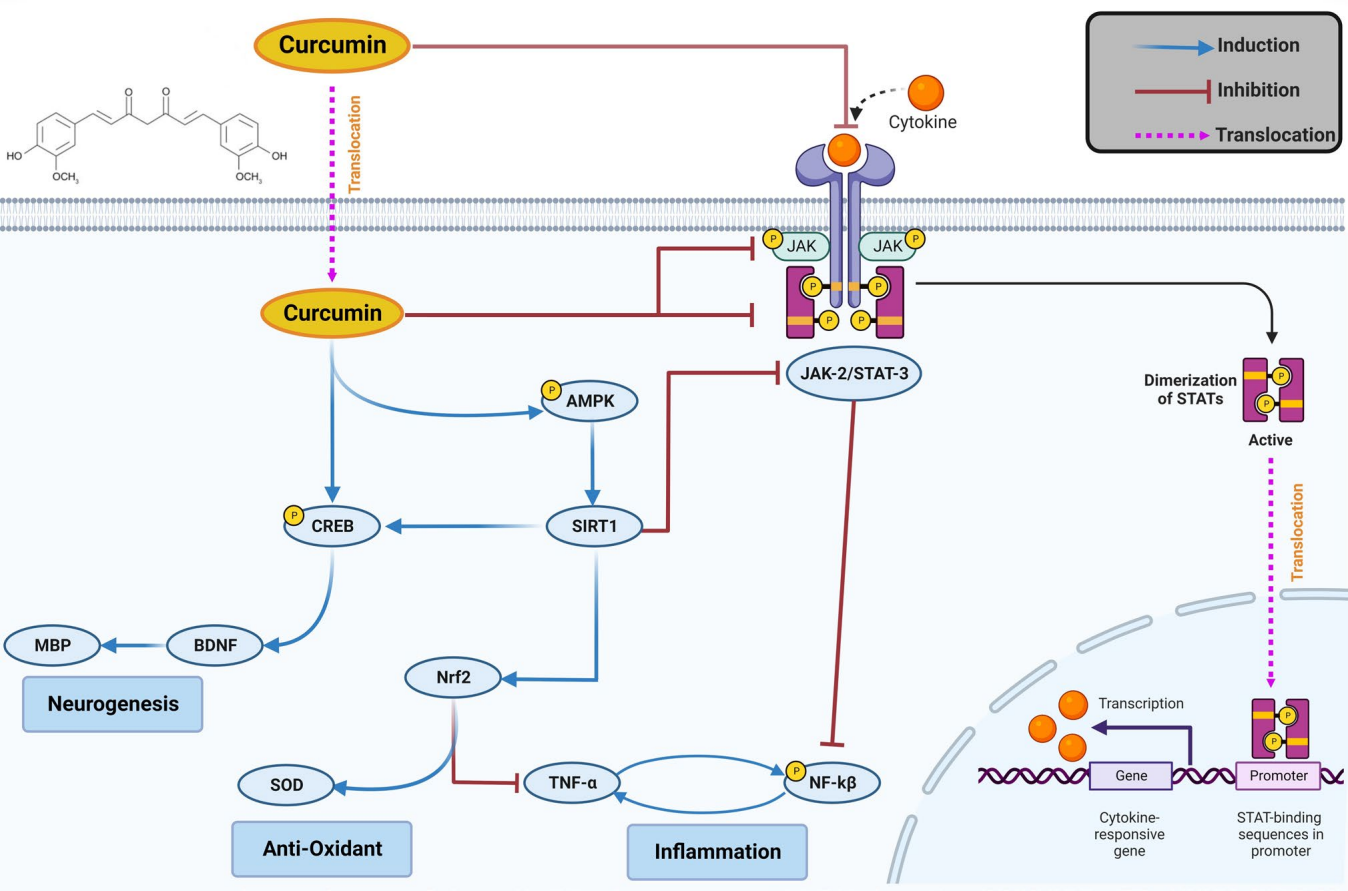

## Introduction

Multiple sclerosis (MS) is a chronic disabling autoimmune disease characterized by hyperactivity of the immune system against the natural myelin protein in the central nervous system (CNS), leading to demyelination and axonal destruction (Cavallo 2020; Dema et al. 2021). MS symptoms drastically differ among patients and involve motor, cognitive, autonomic, and sensory impairments depending on the damaged part of the CNS (Garg and Smith 2015; Gromisch and Dhari 2021). Conspicuously, the prevalence of MS has increased worldwide, imposing a significant financial burden on society, particularly because of the expensive disease-modifying therapies (Hartung 2017; Ysrraelit and Correale 2019; Walton et al. 2020; Yamout et al. 2020). Although several medications are available to control MS relapses, their side effects, high cost, and inability to enhance the remyelination of nerve fibers prompt the need for a novel drug (Bierhansl et al. 2022).

In an attempt to develop a new drug for MS, several studies investigating the pathophysiology of MS have accused oxidative stress and neuroinflammation to trigger MS (Pegoretti et al. 2020; Tobore 2021). Furthermore, researchers have extensively studied the role of adenosine monophosphate (AMP)-activated protein kinase (AMPK) (Paintlia et al. 2013) in the pathophysiology of MS. AMPK is crucial for maintaining the energy balance required for CNS health (Peixoto et al. 2017; Saito et al. 2019). Moreover, it positively modulates silent mating type information regulation 2 homolog 1 (SIRT1), which prevents neurodegeneration, oxidative stress, and neuroinflammation (Costa et al. 2016; Jiao and Gong 2020). Interestingly, increasing evidence suggests that the activation of the AMPK/SIRT1 pathway might regulate MS-induced demyelination, oxidative stress, and neuroinflammation (Paintlia et al. 2013; Elbaz et al. 2018; Dziedzic et al. 2020).

Notably, experimental autoimmune encephalomyelitis, also known as experimental allergic encephalomyelitis (EAE), is a demyelinating and neuroinflammatory animal disease model widely utilized to investigate MS’s pathophysiology and new remedies (Bjelobaba et al. 2018; Burrows et al. 2019). Briefly, autoimmunity against the myelin sheath is mimicked in EAE by actively immunizing the animal with myelin antigens, usually emulsified with an adjuvant that provokes the immune system (Constantinescu et al. 2011). Spinal cord homogenate (SCH) induction is distinguished by the high efficacy and simplicity of induction, in addition to the low cost, even though it is one of several antigens used to induce EAE (Burrows et al. 2019; Sadek et al. 2023). Additionally, SCH induces a relapsing–remitting type of MS, where symptoms peak after 2 weeks of induction, followed by a remitting phase (Lavrnja et al. 2017; Bozic et al. 2018). Indeed, EAE ameliorates cognitive and motor function, as encountered in patients with MS (Rizzo et al. 2021). Noteworthy, previous studies have also supported the role of the AMPK/SIRT1 pathway in EAE pathophysiology (Paintlia et al. 2013; Wang et al. 2016a, b, c).

The interest in using herbal remedies in neurodegenerative diseases has grown dramatically in recent decades with the virtue of their wide margin of safety and multiple pharmacological actions (Wang et al. 2016a, b, c; Velmurugan et al. 2018). Curcumin, a natural polyphenol compound, is known for its antioxidant, anti-inflammatory, antimicrobial, and anticancer properties (Kocaadam and Sanlier 2017; Giordano and Tommonaro 2019). Moreover, multiple studies pointed to curcumin as a promising therapeutic agent in various neuronal diseases, including MS (Qureshi et al. 2018; Mavaddatiyan et al. 2021), Alzheimer’s (Voulgaropoulou et al. 2019), Parkinson’s (Nebrisi 2021), and Huntington’s diseases (Labanca et al. 2021). Despite the extensive study of curcumin in experimental models of MS (Xie et al. 2009; Qureshi et al. 2018; Esmaeilzadeh et al. 2019; I et al. 2022), the influence of curcumin in managing MS cognitive impairment is still vague and needs further study. Surprisingly, Iside et al. shed the light on the ability of multiple natural phytochemicals, including curcumin, to activate AMPK/SIRT1 (Iside et al. 2020). Consequently, we intended to investigate the influence of curcumin against EAE-induced cognitive and motor impairments at the summit of the symptoms. Additionally, we aimed to evaluate the impact of the AMPK/SIRT1 pathway on curcumin’s effect, which was previously unexamined.

## Materials and methods

### Declarations

The experiment followed the Guide for the Care and Use of Laboratory Animals, published by the US National Institutes of Health (Publication No. 85-23, revised 2011), and was approved by the Ethics Committee for Animal Experimentation at the Faculty of Pharmacy, Cairo University (Cairo, Egypt) (permit number: PT-3167). Every attempt was made to protect the animals from suffering during the study.

### Animals

Adult male Swiss–Albino mice aged 6–8 weeks and weighing 20–25 g, were used in the study. Furthermore, adult male Sprague–Dawley rats aged 17–20 weeks weighing 200–250 g, were used to obtain the spinal cord needed for EAE induction. All animals were obtained from the animal facility of the Faculty of Pharmacy, Cairo University (Cairo, Egypt), and were maintained in a soundproof animal house under ideal environmental conditions, including constant temperature (25 °C ± 2 °C), humidity (60% ± 10%), and 12-/12-h light/dark cycle (lights on 6:00 am). The animals were given unrestricted access to a standard chow diet and water ad libitum.

### Drugs and chemicals

Curcumin was obtained from Fisher Scientific International, Inc. (Massachusetts, USA, cat#: AAB2157309, purity: 95.00%). Carboxymethyl cellulose (CMC) and complete Freund’s adjuvant (CFA) were obtained from Sigma-Aldrich Chemical Co (Missouri, USA). Unless mentioned otherwise, all the chemicals were purchased from Sigma-Aldrich Chemical Co (Missouri, USA) and were of the highest analytical grade.

### EAE induction in mice

Spinal cords were extracted from the Sprague–Dawley rats after being euthanized by decapitation under ketamine/xylazine anesthesia (Ko et al. 2019). Then, the extracted spinal cords were emulsified with an equal amount of CFA to prepare the EAE emulsion. EAE was induced in the mice by injecting 100 µL of the prepared emulsion subcutaneously at the base of the mice’s tail on the 1st and 7th days of the experiment (Sadek, Kandil et al. 2023). Similar to the expanded disability status scale that is used to assess MS patients, the EAE clinical scoring system has been created to be used in animals. The mice were examined twice daily for signs of progressive paralysis induced by EAE, and a score was given according to their severity. The scores were graded on a scale of 0–5 (0, normal; 0.5, loss of tonicity in the distal half of the tail; 1, piloerection; 2, entire loss of tail tonicity; 3, paralysis of one hind limb; 4, paralysis of the two hind limbs; or 5, moribund) (Li et al. 2020). The scoring was done by an investigator who was blind to the groups’ identity.

### Experimental design

**Fig. 1:**
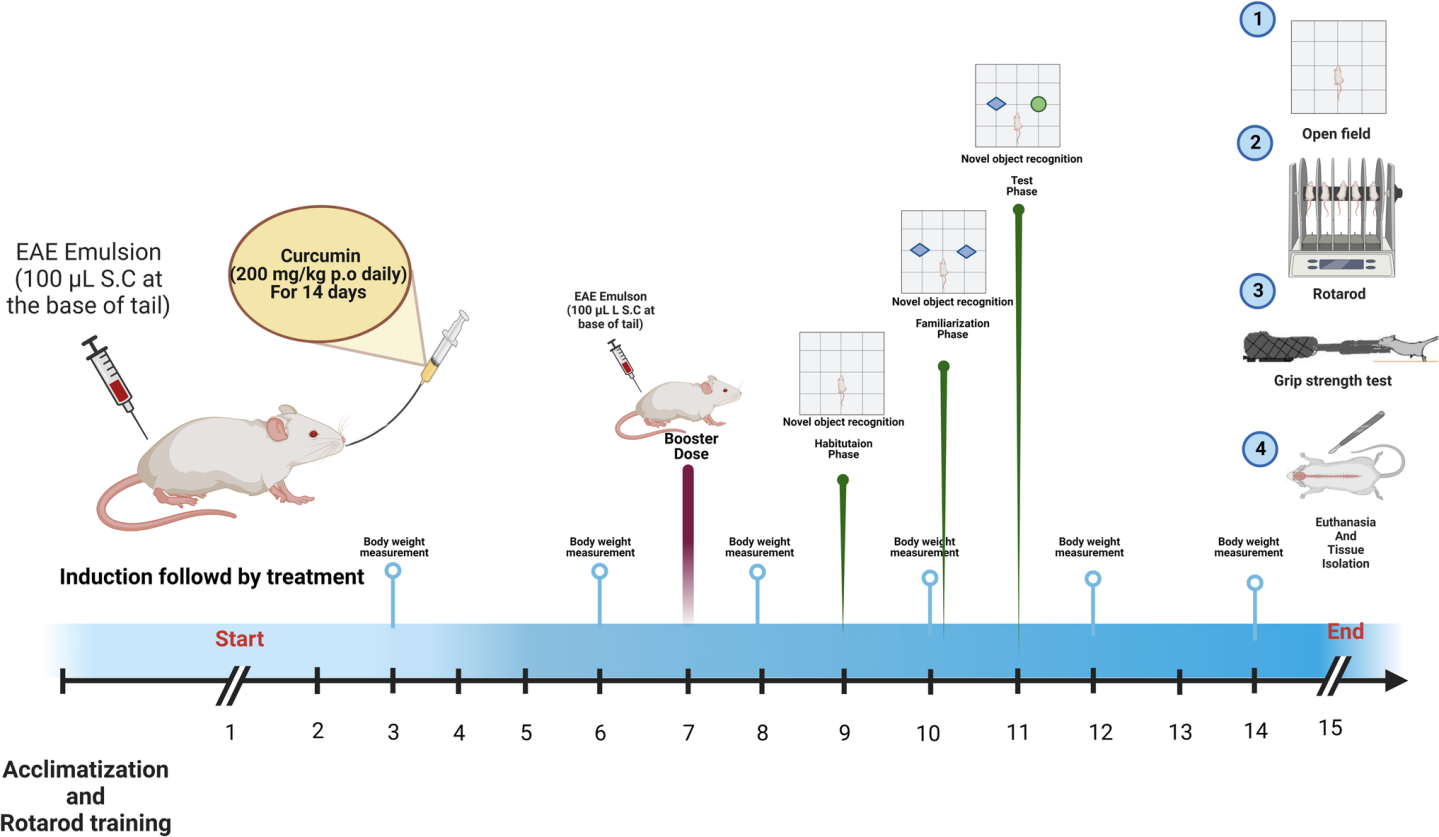
Illustration of the experimental design of the current study used to evaluate the influence of curcumin against EAE-induced MS “Created with BioRender.com.”

The mice were acclimatized for 1 week in the animal house before initiating the experiment. Then, they were randomly distributed into four groups (*n* = 10/group). Group 1 served as the control group and orally received 0.2 ml/day of 0.5% CMC, which is curcumin vehicle. Group 2 animals orally received curcumin (200 mg/kg/day) suspended in 0.5% CMC (Mavaddatiyan et al. 2021). Furthermore, Group 3 mice served as the EAE model group and were injected subcutaneously at the base of the tail with 100 µL of the prepared EAE emulsion on the 1st and 7th days of the experiment, and received 0.2 ml of curcumin vehicle orally. Group 4 mice were subjected to EAE induction, similar to group 3, and were treated with curcumin, similar to group 2. In All groups, the treatments were given 2 h after EAE induction and continued daily for 14 consecutive days, and the total body weight was measured during the study. Noteworthy, all mice belonged to the same cohort (Fig. 1).

### Behavioral tests

We observed impaired cognitive and motor functions with EAE progression, which were assessed using behavioral tests. In this context, the novel object recognition (NOR) test was performed to evaluate the cognitive function on the 11th day where mice with scores > 2 were excluded to prevent the negative interference of motor impairment on the results, consistent with previous studies (Ludwig et al. 2017; Peres et al. 2021). Further, open field, rotarod, and grip strength tests were conducted 24 h after the last curcumin administration, including all mice, to assess motor function, coordination, and strength. The behavioral tests were conducted from the least to the most stressful in a sound-isolated laboratory during the animals’ light cycle to eliminate any variations caused by circadian fluctuations (Yang et al. 2008).

#### Novel object recognition test

Cognitive impairment occurs in the early phases of EAE (Aharoni et al. 2019; Hollinger et al. 2019; Hou et al. 2020). Therefore, the NOR test was conducted between the 9th to 11th day to evaluate cognitive function. Mice with an EAE clinical score > 2 were excluded from the NOR test to avoid false outcomes due to motor dysfunction. The NOR test depends on the distinctive preference of animals for a new object, where the mice memorize the familiar object while they spend more time exploring a new one. The test was performed over three consecutive days. On the 1st day (habituation phase), the mice were allowed to individually acclimate to the apparatus, a wooden box (40 × 40 × 40 cm^3^), for 10 min without any other objects. On the 2nd day (familiarization phase), the mice were placed individually in the middle of the box. They were allowed to explore two identical objects (blue cubes) placed at opposite corners of the box for 10 min. On the 3rd day (test phase), a new object (green cylinder) replaced one of the previous objects (blue cubes), and each mouse was left to explore the objects in the box for 3 min. Objects were made of wood, and the animals could not displace them. Objects had smooth, bright surfaces and were rinsed with 70% ethanol after each test to avoid bias due to odors left by the preceding mouse. Exploration was considered when the animal directed its nose at a distance of 2 cm from the object, while sitting on the object was not considered exploration. The test was recorded on video to analyze the preference index, which is the time the animal spent investigating the novel object divided by the whole exploration time. We also evaluated the discrimination index, the difference in time spent exploring both objects (the novel object and the familiar object) divided by the total time of exploration (El Sayed et al. 2021).

#### Open field test

The open field test was conducted in a square wooden box (40 × 40 × 25 cm^3^) with red walls and a polished black floor, separated by white lines into 16 equal squares. Each mouse was allowed to freely explore the box for 3 min. The test was conducted in a quiet atmosphere illuminated by a dim light, and the mice were carefully moved to and from the box to prevent any stress. The box was cleaned with 70% alcohol after each mouse to remove any odors. Any-Maze video tracking software (Version 7.1, Stoelting Co, Illinois, USA) was used to assess the following parameters for each mouse; the total distance crossed (meter (m)), mean speed (m/sec), ambulation frequency (number of lines crossed by the mouse), rearing frequency (number of times the mouse stood on its hind limb without forelimb support), and immobility time (the time passed in a sec without any movement) (Walsh and Cummins 1976).

#### Rotarod test

Motor coordination was examined using a 3-cm diameter rod rotating at 25 rpm, and raised above a cushion by 30-cm to discourage the animal from jumping. The mice were trained for three consecutive days before starting the experiment to stay on the rotating rod for 5 min; then, on the test day, the latency to fall off the rotarod was recorded in a sec with a cutoff at 300 s (Jones and Roberts 1968).

#### Grip strength test

To assess the grip strength of the mice, we used a grip strength meter (Model 47200, Ugo Basile, Comerio, Italy) where each mouse was placed horizontally in front of a grasping bar, and its tail was slowly and gently pulled back until the mouse releases its grip. The test was repeated thrice, and the average was considered the grip strength expressed in gram force (gf) (Ge et al. 2016).

### Brain processsing

A day after the behavioral tests, the mice were euthanized by decapitation under ketamine/xylazine anesthesia (Ko, Mulia et al. 2019), and their brains were quickly dissected, washed with ice-cold saline, and weighed. After that, the brains were randomly divided into two subsets, where brains of the first subset (*n* = 4/group) were fixed in 10% (v/v) formalin for histological examination, while brains of the second subset (*n* = 6/group) were cut into two equal longitudinal halves which were flash-frozen in liquid nitrogen, and kept at − 80 °C for future biochemical tests.

### Biochemical parameters

#### Enzyme-linked immunosorbent assay (ELISA)

The first halves of the second subset brains were homogenized in phosphate buffer saline to prepare 10% (w/v) homogenates that were used to assess SIRT1, brain-derived neurotrophic factor (BDNF), myelin basic protein (MBP), p65-nuclear factor-kappa beta (p65-NF-kβ), and tumor necrosis factor-alpha (TNF-α) using ELISA assay kits from Elbascience (Texas, USA, cat#: E-EL-M0350, cat#: E-EL-M0203, cat#: E-EL-M0805, cat#: E-EL-M0838, cat#: E-EL-M3063, respectively). Similarly, the nuclear factor erythroid 2–related factor 2 (Nrf2) levels and the superoxide dismutase (SOD) activities were determined in the brain homogenates with ELISA kits from My BioSource (California, USA, cat#: MBS2516218 and cat#: MBS034842, respectively). The tests were performed according to the manufacturer’s instructions. The results were expressed as pg/mg protein for BDNF and TNF-α, ng/mg protein for SIRT1, MBP, and Nrf2, and U/mg protein for SOD, where the protein content of tissue homogenate was determined by the Lowry method (Lowry et al. 1951).

#### Western blot analysis

The second halves of the second subset brains were homogenized in radioimmunoprecipitation assay buffer (25 mM Tris–HCl, 150 mM NaCl, 1% NP-40, 1% sodium deoxycholate, 0.1% SDS, pH 7.6) containing protease and phosphatase inhibitor cocktail (ThermoFisher Scientific, Massachusetts, USA). Then, the homogenates were constantly agitated for 1 h at 4 °C and centrifuged for 30 min at 12,000 rpm at 4 °C. The supernatants were aspirated, and the total protein content was determined using a bicinchoninic acid protein measurement kit (ThermoFisher Scientific, Massachusetts, USA, Cat#: 23225). Then, an equal amount of protein from each sample (30 µg) was separated by sodium dodecyl sulfate–polyacrylamide gel electrophoresis and then transferred to a polyvinylidene difluoride membrane that was blocked using 5% bovine serum albumin for 1 h at room temperature. Subsequently, the membranes were incubated with primary antibodies against p-AMPK-α1,2^Thr172^ (1:1000 cat#: PA5-37821), p-cyclic AMP response element-binding protein (p-CREB)^Ser133^ (1:1000, cat#: PA5-85645), p-Janus kinase 2 (p-JAK2)^Tyr1007, Tyr1008^ (1:1000, cat#:44-426G), p-signal transducers and activators of transcription 3 (p-STAT3)^Tyr705^ (1:1000, cat#: 44-380G), and beta-actin (1:1000, cat#: PA1-183) (ThermoFisher Scientific, Massachusetts, USA) overnight on a roller shaker at 4°C. After washing, the membranes were incubated with the peroxidase-labeled secondary antibodies at room temperature for 1 h. Finally, the blots were visualized with an enhanced chemiluminescence detection reagent (Amersham Biosciences, New Jersey, USA). Protein was quantified by densitometric analysis using a scanning laser densitometer (GS-800 system, Bio-Rad, California, USA). The results were expressed as arbitrary units after normalization for beta-actin expression.

### Histopathological examination

The brains of the first subset were promptly fixed in 10% formalin after scarification for 24 h. After washing with ice-cold saline, the brains were dehydrated in serial dilutions of alcohol and embedded in paraffin blocks. Five-micron slices were sectioned and stained with hematoxylin and eosin (H & E) to examine any morphological changes and signs of inflammatory cell infiltration in the hippocampal CA3 region and the corpus callosum. The corpus callosum is a dense myelinated fiber bundle in the central nervous system and its demyelination occurs early in MS (Rimkus Cde et al. 2011). Herein, the severity of the hippocampus damage was further assessed via toluidine blue staining, where the number of intact neurons was determined. Moreover, the corpus callosum’s demyelination was assessed using luxol fast blue (LFB) staining. The percentage of corpus callosum’s myelinated nerve fibers and the number of intact neurons in the CA3 of the hippocampus were quantified using a full HD microscope camera and the Leica application module for tissue section analysis (Leica Microsystems GmbH, Wetzlar, Germany) (Abd El Aziz et al. 2021). The histopathological examination was performed by an expert investigator who was blinded to the identity of the samples.

### Statistical analysis

Normality and homogeneity of the data were first tested by Shapiro–Wilk and Brown–Forsythe tests, respectively. Parametric data were analyzed using one-way analysis of variance (ANOVA) followed by the Tukey post hoc test. The results were expressed as mean ± standard deviation (SD). A two-way ANOVA followed by the Tukey post hoc test was used in body weight analysis, where both time and treatment affect the result. Nonparametric data were tested by Kruskal–Wallis test followed by Dunn’s post hoc test and expressed as median ± range. GraphPad Prism software (Version 9, San Diego, California, USA) was used to perform the analysis. A probability (*p*) < 0.05 was set as the significance limit for all comparisons.

## Results

Noteworthy, data exported from both the control group and the curcumin-only group were statistically similar in all measured tests.

### Curcumin alleviated the clinical score of EAE and EAE-induced reduction in total body

**Fig. 2:**
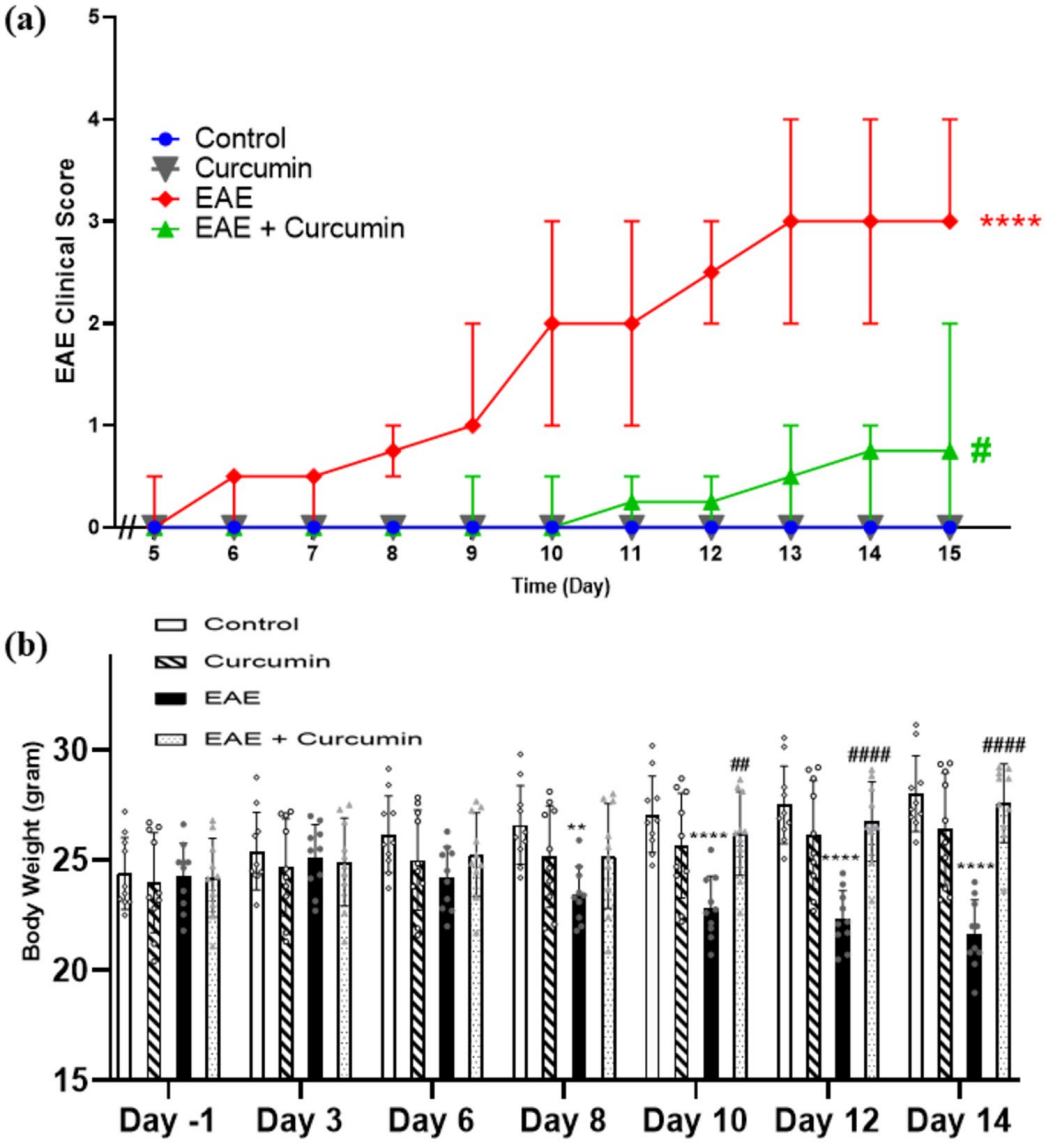
Effect of curcumin on **a** EAE clinical score and **b** total body weight. In **a** values are represented as median ± range (*n* = 10/group). **** statistically significant from the control group at *p* < 0.0001, ^#^ statistically significant from the EAE group at *p* < 0.05, using Kruskal–Wallis test followed by Dunn’s post hoc test. In (b) values are represented as the mean ± SD (*n* = 10/ group). ** significantly different from the control group at p < 0.01, **** significantly different from the control group at *p* < 0.0001, ^####^ significantly different from the EAE group at p < 0.0001, using two-way ANOVA followed by Tukey’s post hoc test

Initial evaluation of the possible therapeutic effect of curcumin in the EAE model was done using general observation and the EAE clinical scoring. The EAE group showed higher clinical scores (median = 3, on day 15) compared to the control group (test statistic = 34.41, *p* < 0.0001), which were significantly reduced by daily administration of curcumin (median = 0.75, at day 15) compared to the EAE mice (*p* < 0.05) (Fig. 2a).

Inline, EAE triggered a weight reduction in mice as it negatively affected food intake. Herein, EAE mice showed a 22.74% body weight reduction (*F* (18, 216) = 49.43) compared to the control group, on day 14 (*p* < 0.0001). On the other hand, the curcumin remedy reversed this reduction and showed a notable increase in total body weight by 1.27-fold, compared to the EAE group, on day 14 (*p* < 0.0001) (Fig. 2b).

### Curcumin diminished EAE-induced cognitive impairment

**Fig. 3:**
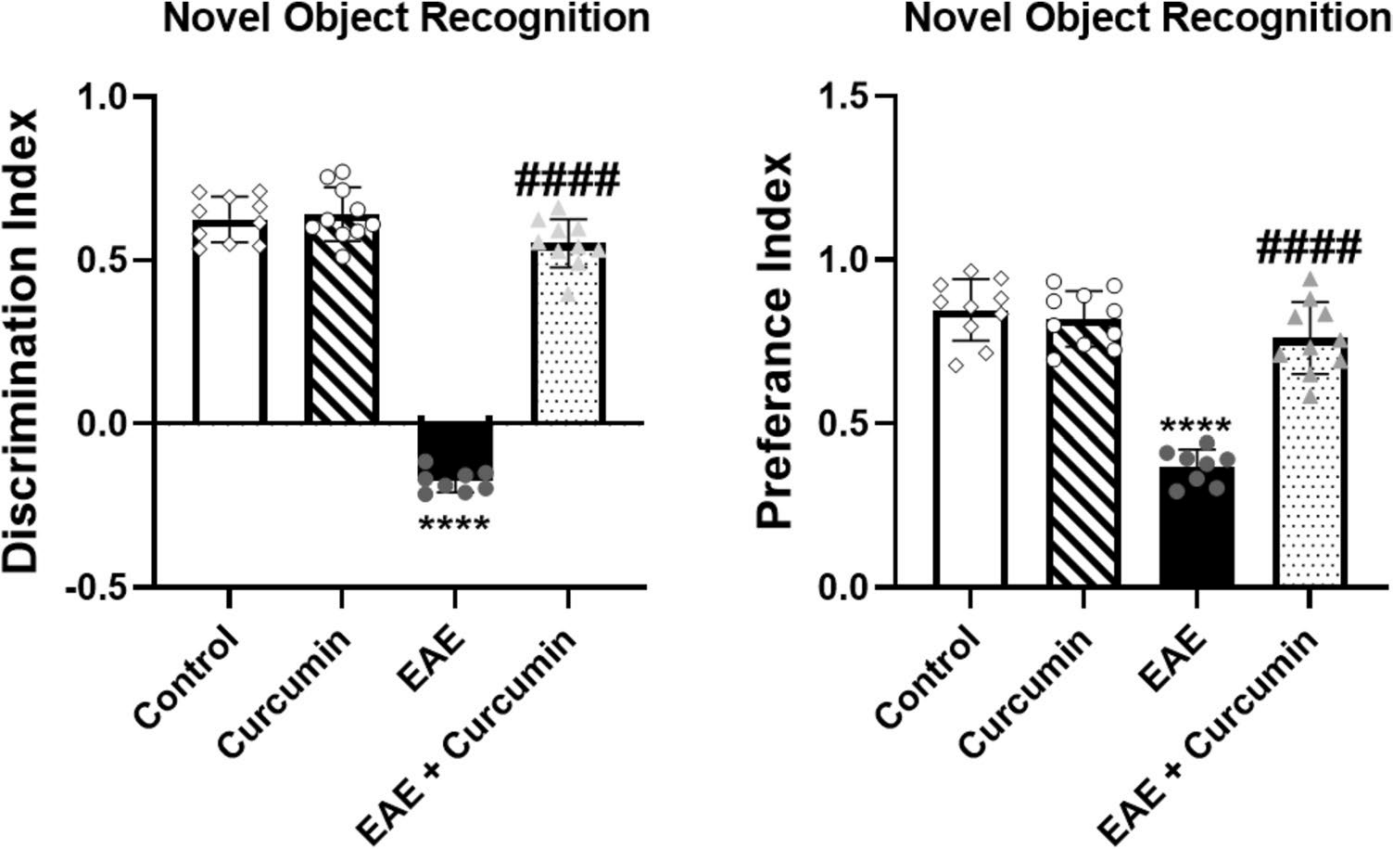
Curcumin diminished EAE-induced cognitive impairment. Values are represented as the mean ± SD (*n* = 10/ group, except EAE group, where (*n* = 8). **** significantly different from the control group at *p* < 0.0001, ^####^ significantly different from the EAE group at *p* < 0.0001, using one-way ANOVA followed by Tukey’s post hoc test

NOR test was used to evaluate the cognitive impairment induced by EAE. The EAE mice showed significantly reduced cognition as evidenced by a decrease in the discrimination and preference indices in the NOR test (*F* (3, 34) = 270.30 and 52.40, respectively) by 128.00% and 56.49%, respectively, compared to the control group (*p* < 0.0001). However, curcumin treatment ameliorated this deterioration by causing a 3.16- and 2.07-fold increase in both indices, respectively, compared to the EAE mice (*p* < 0.0001) (Fig. 3).

**Fig. 4:**
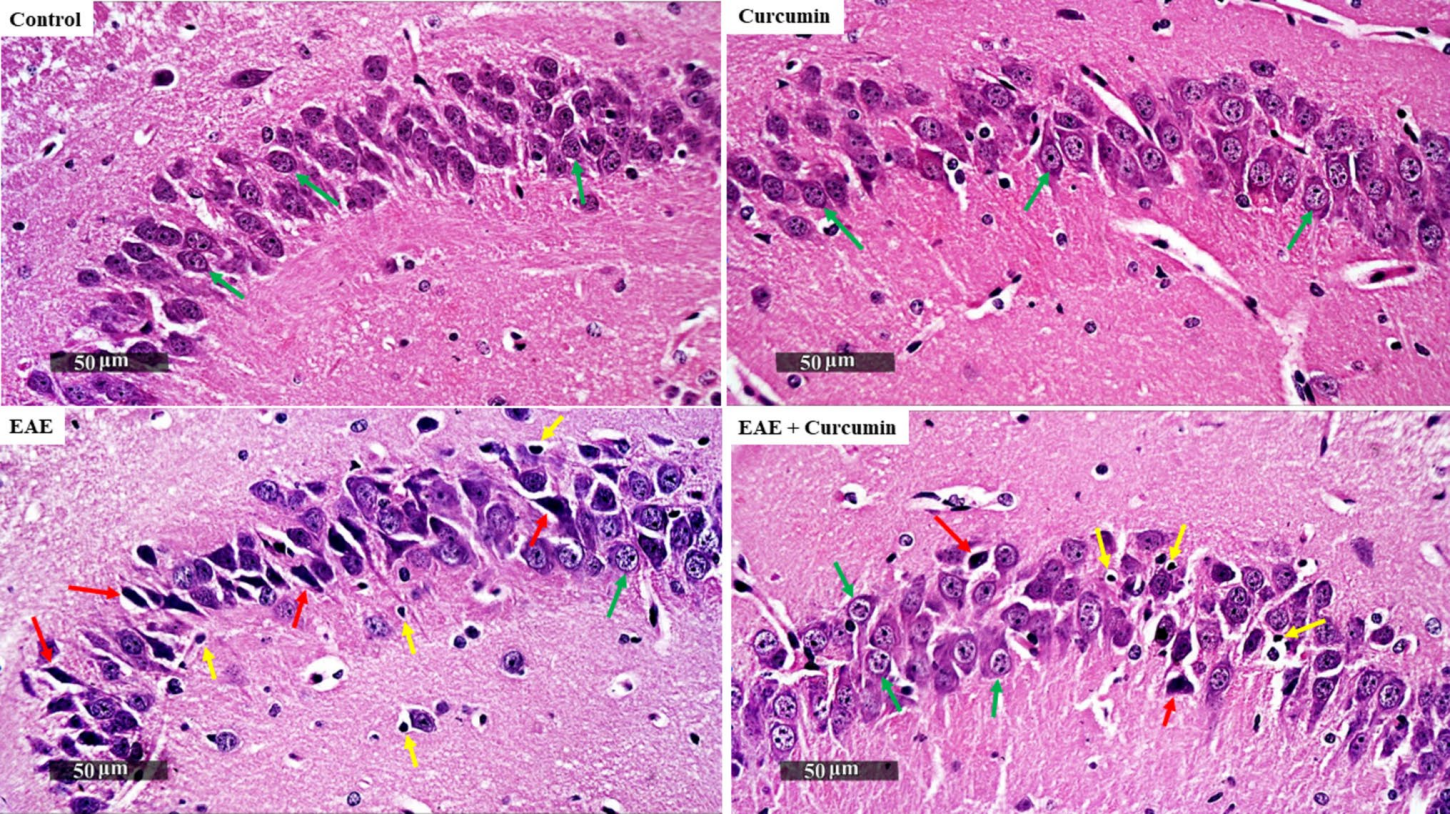
Curcumin alleviated EAE-induced histological alterations in the hippocampus stained by H&E (× 400 Magnification, *n* = 4/group). Control and curcumin group revealed intact neurons (green arrows). EAE showed high quantity of shrunken degenerated neurons (red arrows) with infiltration of microglia (yellow arrows). Curcumin-treated mice revealed a few records of neuronal damage and microglia infiltration with higher records of normal neurons

Since the hippocampus has a crucial role in cognition, the H & E-stained CA3 region of the hippocampus was microscopically examined. The control group showed normal organized morphological features of the hippocampal layers with apparent intact pyramidal neurons demonstrating distinct nuclear and subcellular details with an intact intercellular matrix. Conversely, the EAE group showed minimal intact pyramidal neurons with abundant shrunken, pyknotic degenerated neurons with indistinct subcellular details, mild perineuronal edema, and an abundance of reactive microglial cell infiltration. Interestingly, curcumin exerted a neuroprotective effect, as shown by the normal appearance of the brain matrix with minor neuronal damage and reactive glial cell infiltrates (Fig. 4).

**Fig. 5:**
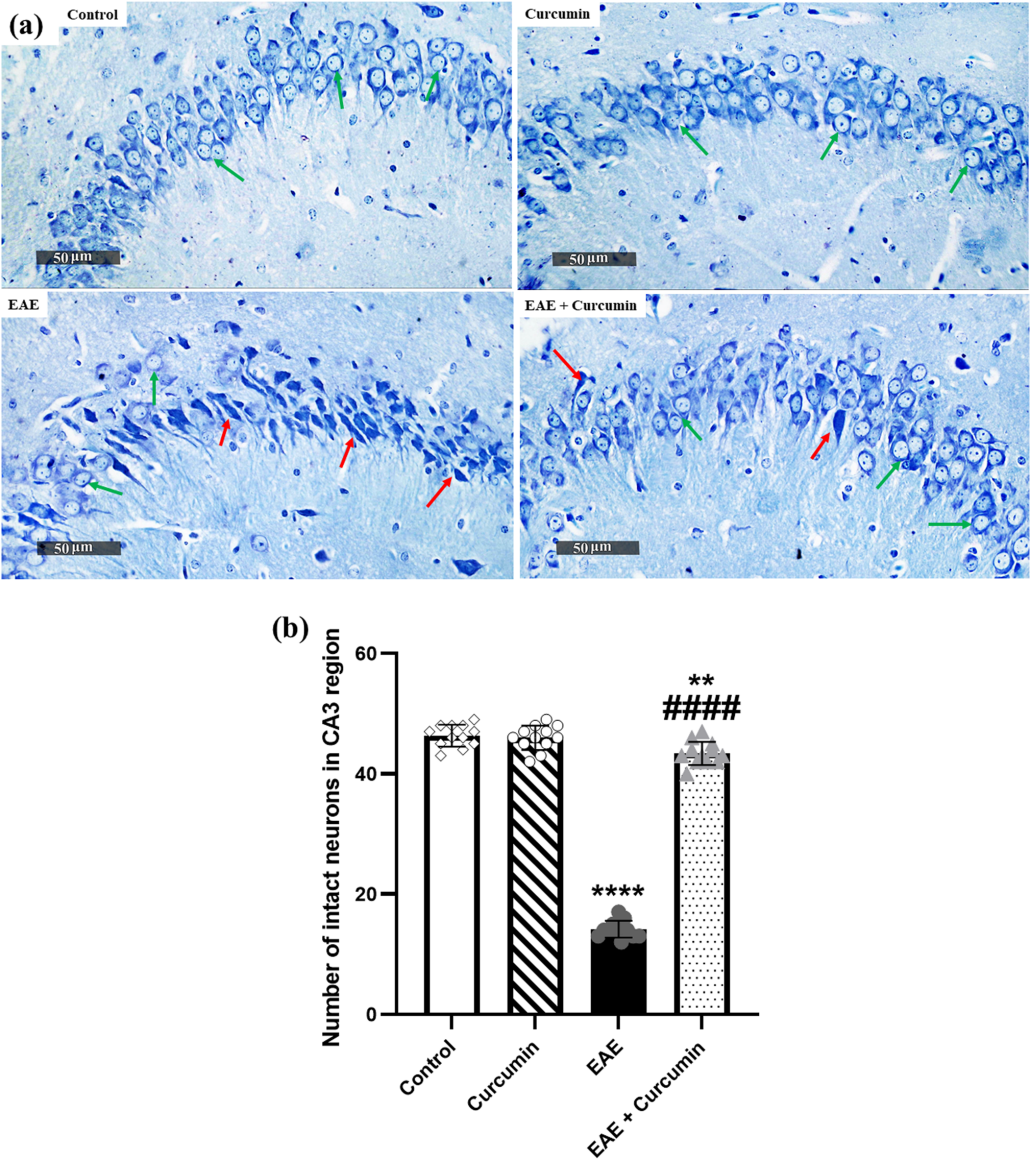
Curcumin alleviated EAE-induced histological alterations in the toluidine blue-stained CA3 region of the hippocampus (× 400 Magnification, *n* = 4/group). **a** Representative histological figures of the CA3 region of the hippocampus stained by toluidine blue. Control and curcumin group revealed normal intact neurons (green arrows), while EAE mice showed degenerated neurons (red arrows). Curcumin-treated mice revealed more normal pyramidal neurons, compared to the EAE mice. **b** Number of intact neurons in the CA3 region of the hippocampus, stained by toluidine blue staining. Values are represented as the mean ± SD (*n* = 4/ group). ** significantly different from the control group at *p* < 0.01, **** significantly different from the control group at *p* < 0.0001, ^####^ significantly different from the EAE group at *p* < 0.0001, using one-way ANOVA followed by Tukey’s post hoc test

Inline, the CA3 region of the hippocampus, stained with toluidine blue, was examined microscopically to further evaluate and quantify the intactness of hippocampal neurons. EAE mice showed a 69.42% reduction in the number of intact neurons, compared to the control groups (*F* (3, 44) = 884.20, *p* < 0.0001). Contrarily, the curcumin group showed a 3.06-fold increase in the number of intact neurons (*p* < 0.0001), compared to the EAE groups (Fig. 5a, b).

### Curcumin minimized EAE-induced motor dysfunction

EAE caused motor impairment and muscular weakness, as evidenced by the results of the open field, rotarod, and grip strength behavioral tests. EAE reduced the total distance, mean speed, ambulation, and rearing frequencies in the open field test (*F* (3, 36) = 75.88, 47.80, 60.56, and 37.60, respectively) by 77.17%, 69.08%, 65.36%, and 76.77%, respectively, compared to the control (*p* < 0.0001). Curcumin treatment increased the aforementioned parameters by 3.33-, 2.40-, 2.36-, and 3.09-fold, respectively, compared to the EAE group (*p* < 0.0001). Moreover, EAE increased the immobility time (*F* (3, 36) = 90.38) in the open field by 4.12-fold compared to the control, while curcumin decreased it by 56.26% compared to the EAE mice (*p* < 0.0001).

**Fig. 6:**
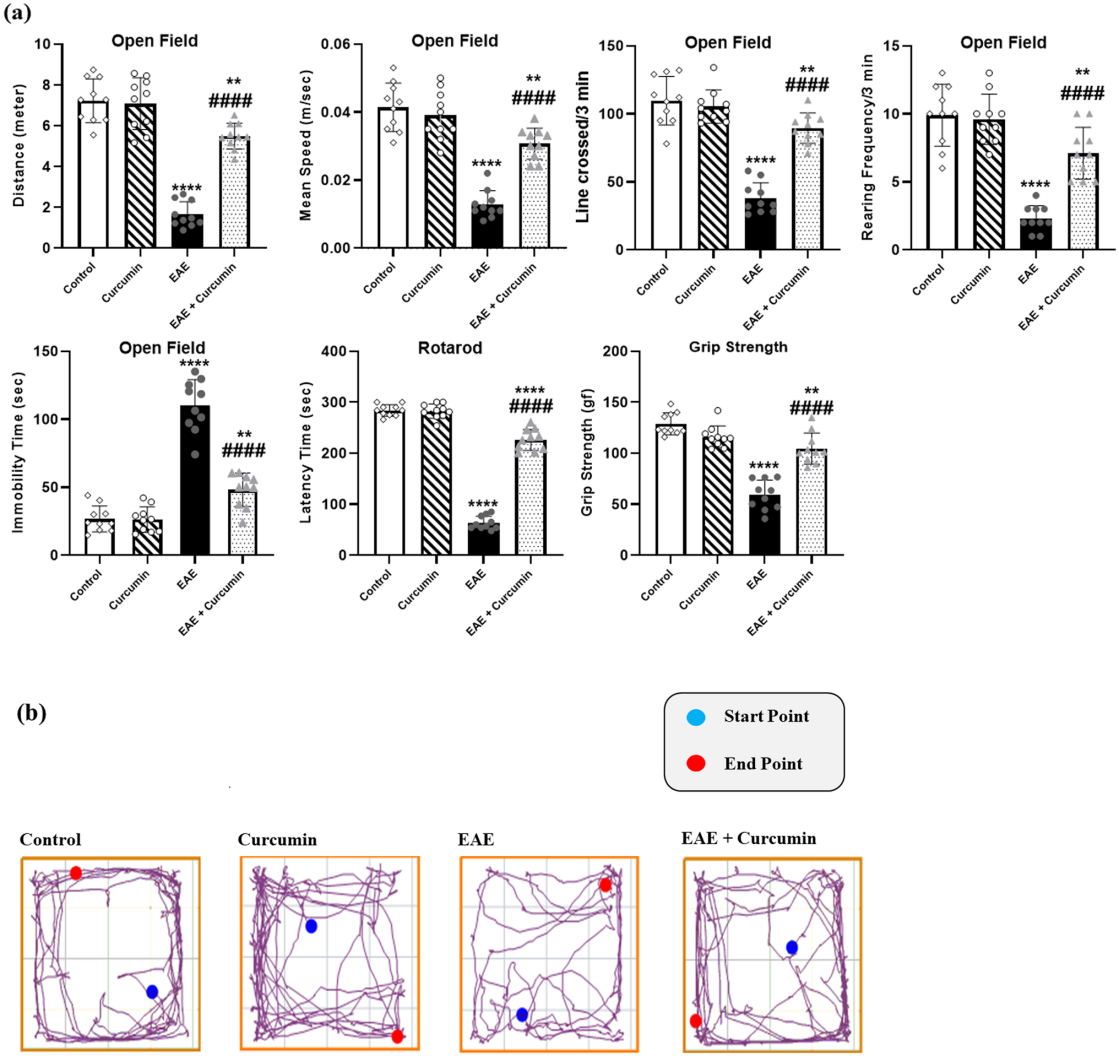
Curcumin minimized EAE-induced motor dysfunction. **a** Motor behavioral tests. **b** Open field track plots exported from Any-Maze video tracking software. Values are represented as the mean ± SD (*n* = 10/ group). ** significantly different from the control group at *p* < 0.01, **** significantly different from the control group at *p* < 0.0001, ^####^ significantly different from the EAE group at *p* < 0.0001, using one-way ANOVA followed by Tukey’s post hoc test

Similarly, EAE reduced the time spent by the mice on the rotarod and its grip strength (*F* (3, 36) = 468.90 and 54.52, respectively) by 77.65% and 54.2%, respectively, compared to the control group (*p* < 0.0001). Curcumin treatment elevated these parameters by 3.56-fold, and 77.17%, respectively, compared to the EAE group (*p* < 0.0001) (Fig. 6).

### Curcumin altered EAE-induced changes in the p-AMPK^Thr172^ and SIRT1 protein levels

**Fig. 7:**
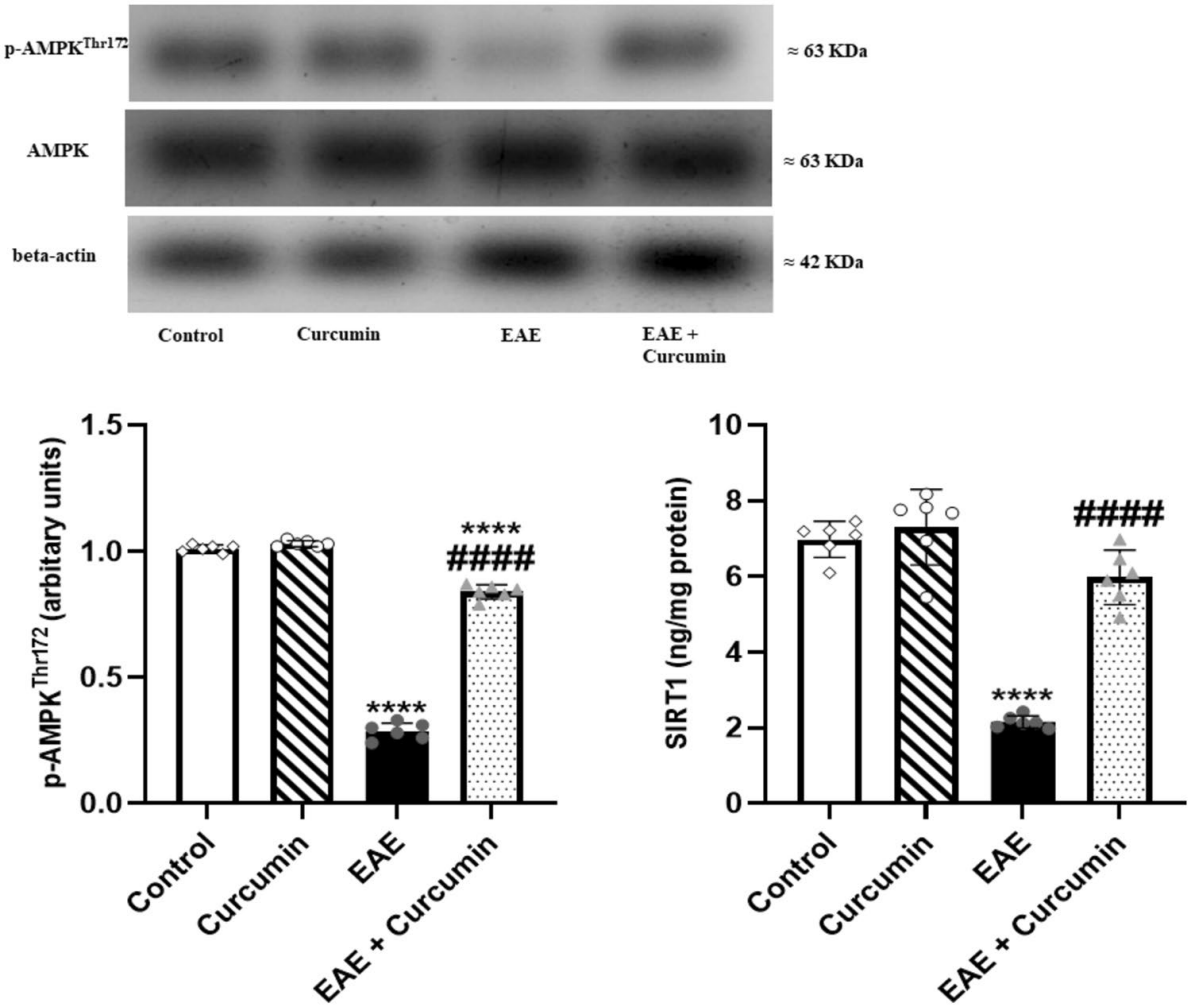
Curcumin altered EAE-induced changes in p-AMPK^Thr172^ and SIRT1 protein levels. Notably, p-AMPK^Thr172^ levels were determined using western blotting technique, while SIRT1 levels were quantified using ELISA technique. Values are represented as the mean ± SD (*n* = 6/ group). **** significantly different from the control group at *p* < 0.0001, ^####^ significantly different from the EAE group at *p* < 0.0001, using one-way ANOVA followed by Tukey’s post hoc test

Protein expression levels of p-AMPK^Thr172^ and SIRT1 were assessed to explore the effect of curcumin on the AMPK/SIRT1 pathway. EAE significantly reduced p-AMPK^Thr172^ and SIRT1 levels (*F* (3, 20) = 1286.00, 76.21, respectively) by 71.67% and 69.09%, respectively, compared to the control (*p* < 0.0001). However, curcumin activated this pathway, as shown by the elevation of p-AMPK^Thr172^ and SIRT1 levels by 2.93- and 2.77-fold, respectively, compared to the EAE mice (*p* < 0.0001) (Fig. 7).

### Curcumin ameliorated EAE-mediated demyelination and neurodegeneration

**Fig. 8:**
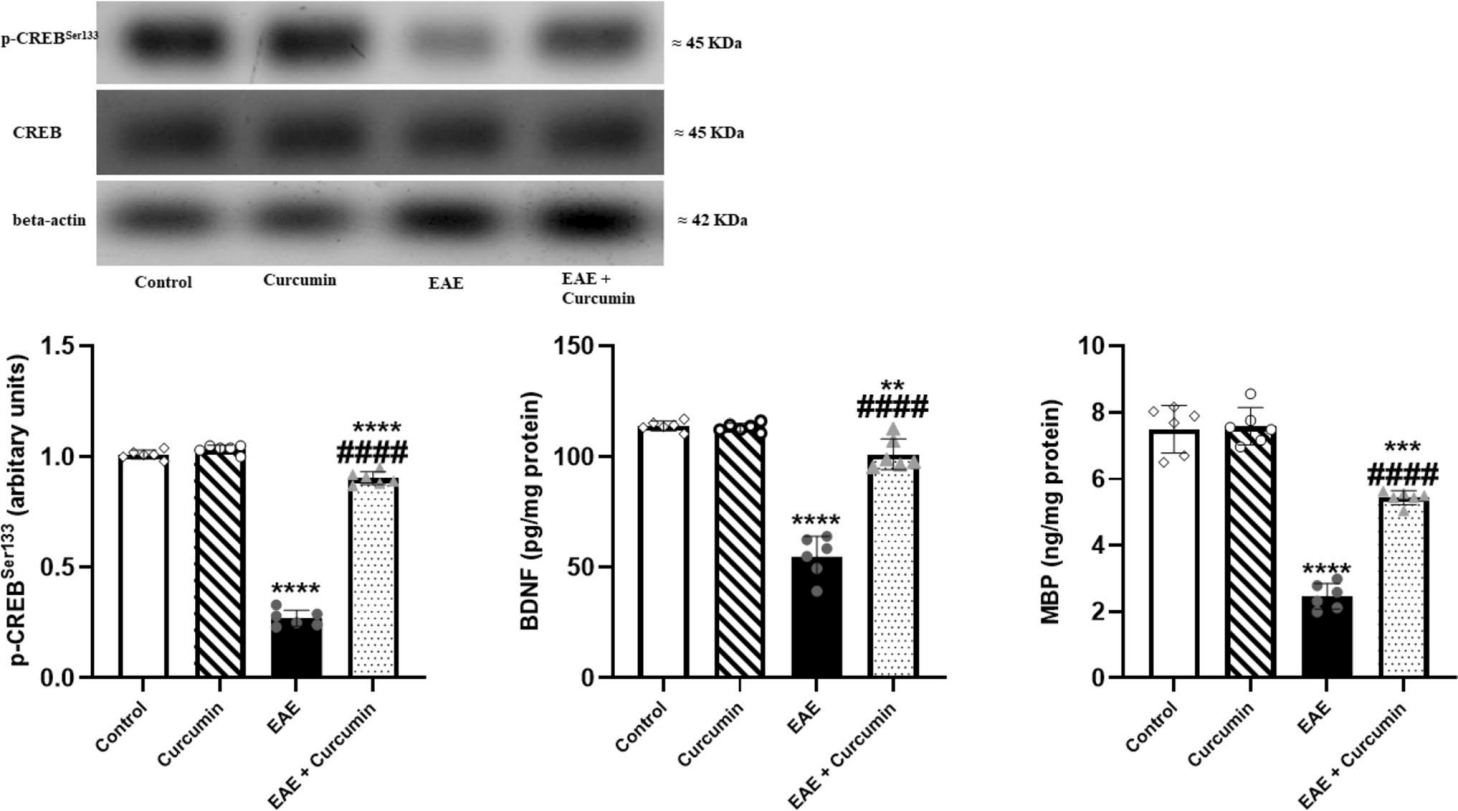
Curcumin ameliorated EAE-induced demyelination and neurodegeneration. Notably, p- CREB^Ser133^ levels were determined using western blotting technique, while BDNF and MBP levels were quantified using ELISA technique. Values are represented as the mean ± SD (*n* = 6/ group). ** significantly different from the control group at *p* < 0.01, *** significantly different from the control group at *p* < 0.001, **** significantly different from the control group at *p* < 0.0001, ^####^ significantly different from the EAE group at *p* < 0.0001, using one-way ANOVA followed by Tukey’s post hoc test

We investigated the CREB/BDNF/MBP pathway to evaluate the myelin sheath as demyelination is a hallmark of MS. EAE suppressed the levels of p-CREB^Ser133^, BDNF, and MBP (*F* (3, 20) = 1036.00, 131.40, and 134.90, respectively, *p* < 0.0001) by 73.27%, 51.90%, and 67.09%, respectively, compared to the control group. Contrarily, curcumin activated CREB/BDNF/MBP pathway and boosted their levels by 3.35-, 1.84-, and 2.21-fold, respectively, compared to the EAE group (*p* < 0.0001) (Fig. 8).

**Fig. 9:**
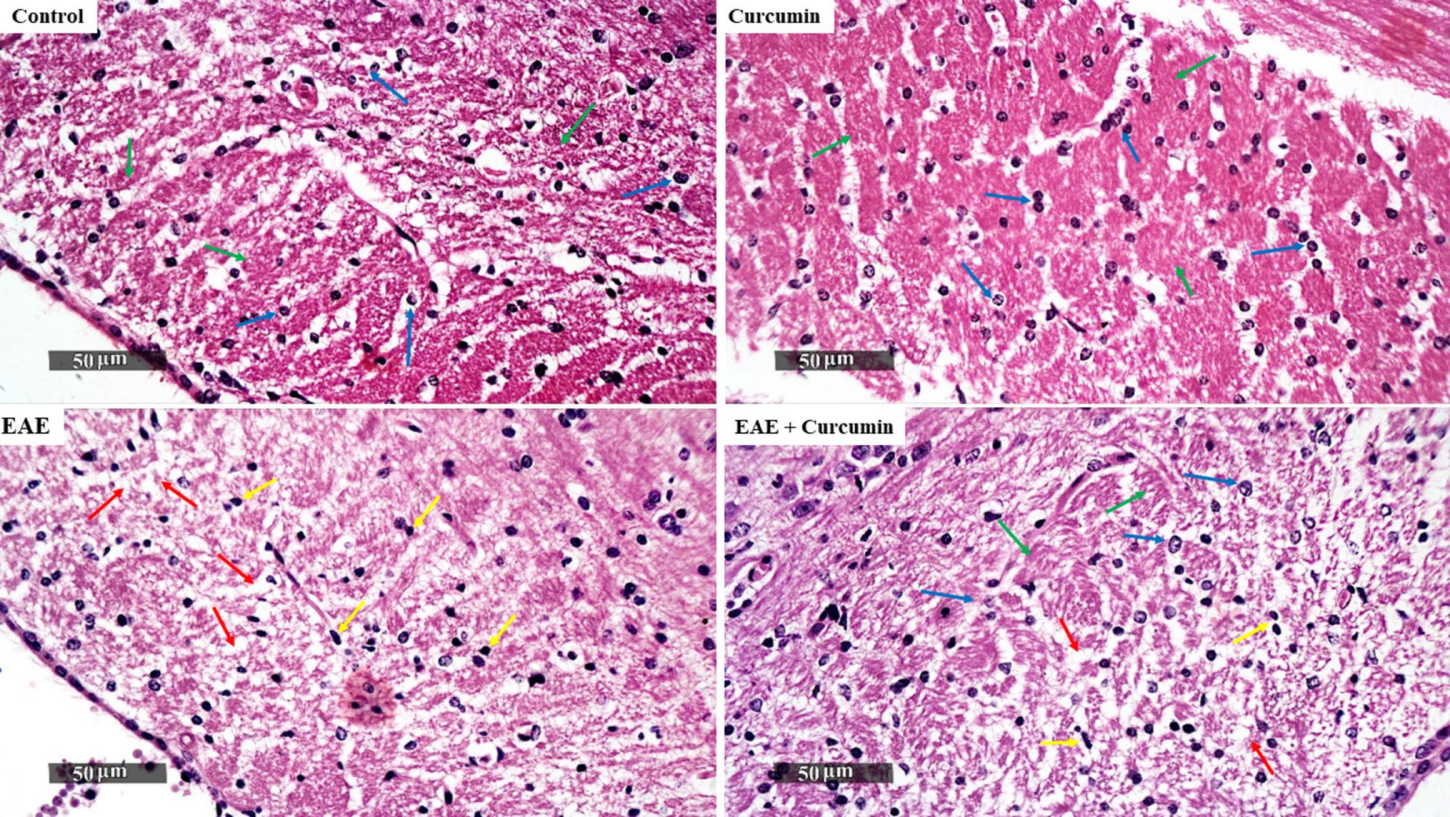
Curcumin alleviated EAE-induced histological alterations in the corpus callosum stained by H&E (× 400 Magnification, *n* = 4/group). Control and curcumin group showed normal structures of the corpus callosum region (green arrows) and normal oligodendrocyte (blue arrows), whereas EAE mice showed disorganized myelinated nerve fibers (red arrows) with microglia infiltration (yellow arrows). In contrast to EAE mice, treated mice showed higher records of healthy oligodendrocytes and lower records of microglia

In addition to investigating the role of CREB/BDNF/MBP in determining the myelin sheath condition, we examined the corpus callosum, a highly myelinated brain region, to evaluate the treatment efficacy. Initially, the H & E-stained sections of the normal mice demonstrated normally organized histological structures of the corpus callosum with abundant densely packed myelinated nerve fibers and normally organized oligodendrocytes with slight abnormal infiltrates. Conversely, EAE mice showed a focal area of disorganized myelinated nerve fibers with significant vacuolization in the rostral body zone of the corpus callosum, associated with oligodendrocytic loss and abundant reactive microglial infiltrates. Remarkably, curcumin treatment distinctly ameliorated EAE-induced alterations and significantly restored the myelinated nerve fibers’ densities and oligodendrocytes together with moderate reactive microglial cell infiltrates and minor vacuolization of white matter (Fig. 9).

**Fig. 10:**
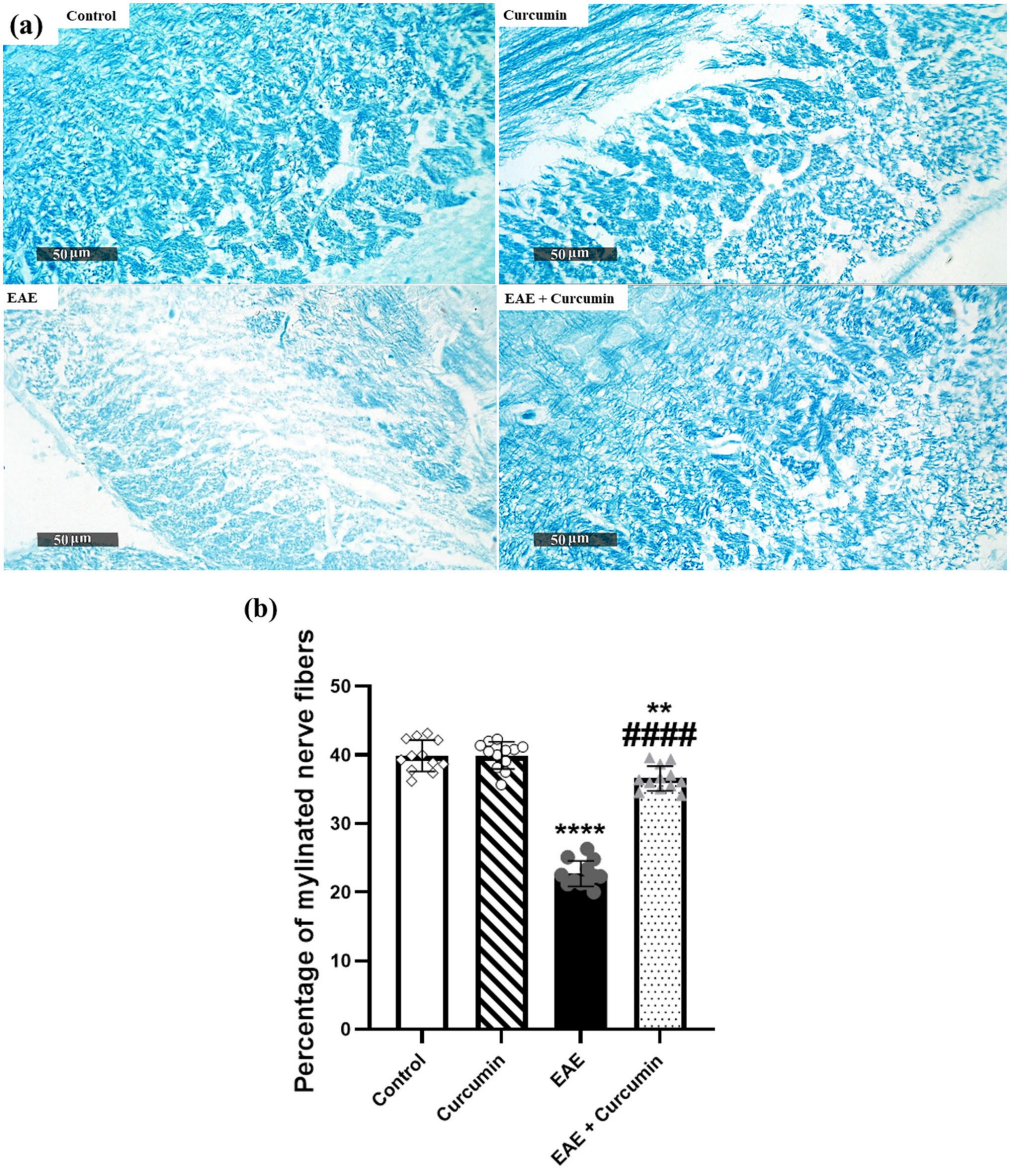
Curcumin alleviated EAE-induced histological alterations in the corpus callosum stained by LFB (× 400 Magnification, *n* = 4/group). **a** Representative histological figures of the corpus callosum stained by LFB. Control and curcumin group revealed myelinated normal neurons with normal stain intensity, while EAE mice revealed a reduction in LFB stain intensity, reflecting disorganized myelinated nerve fibers. Treated mice revealed more myelinated neurons, as compared to the EAE mice. **b** Percentage of myelinated nerve fibers in the LFB images. Values are represented as the mean ± SD (*n* = 4/ group). ** significantly different from the control group at *p* < 0.01, **** significantly different from the control group at *p* < 0.0001, ^####^ significantly different from the EAE group at *p* < 0.0001, using one-way ANOVA followed by Tukey’s post hoc test

Furthermore, we performed LFB staining to investigate curcumin’s effect in preserving the myelin sheath in the corpus callosum. Our findings revealed a noticeable decrease in LFB staining intensity with a markedly reduced percentage of myelinated nerve fibers in the LFB images (43.11%) and significant vacuolization in the EAE group compared with the control groups (*F* (3, 44) = 203.10, *p* < 0.0001). However, the curcumin group exhibited an apparent increase in the percentage of myelinated nerve fibers by 62.14% compared with the EAE group (*p* < 0.0001), indicating its neuroprotective effect in the EAE model (Fig. 10a, b).

### Curcumin alleviated EAE-induced oxidative stress

**Fig. 11:**
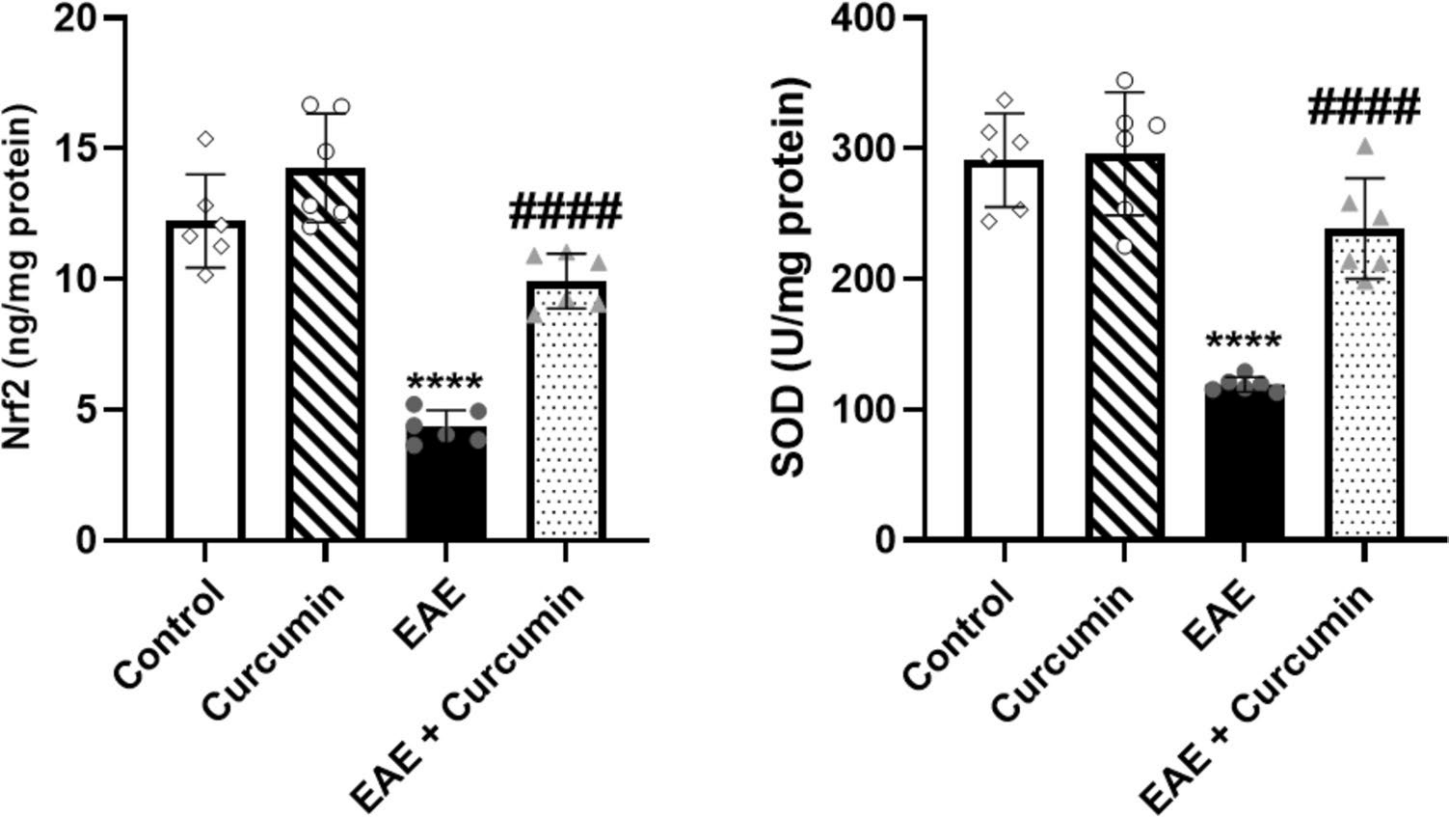
Curcumin amended EAE-induced oxidative stress. Notably, Nrf2 and SOD levels were quantified using ELISA technique. Values are represented as the mean ± SD (*n* = 6/ group). **** significantly different from the control group at *p* < 0.0001, ^####^ significantly different from the EAE group at *p* < 0.0001, using one-way ANOVA followed by Tukey’s post hoc test

EAE induction triggered oxidative stress in the brain as shown by the decline in Nrf2 level and SOD activity (*F* (3, 20) = 48.75 and 32.28, respectively) by 64.46% and 59.04%, respectively, compared to the control (*p* < 0.0001). Contrastingly, curcumin displayed an antioxidant effect as indicated by the increase in Nrf2 level and SOD activity by 2.28- and 2.00-fold, respectively, compared to the EAE mice (*p* < 0.0001) (Fig. 11).

### Curcumin altered EAE-induced neuroinflammation

**Fig. 12:**
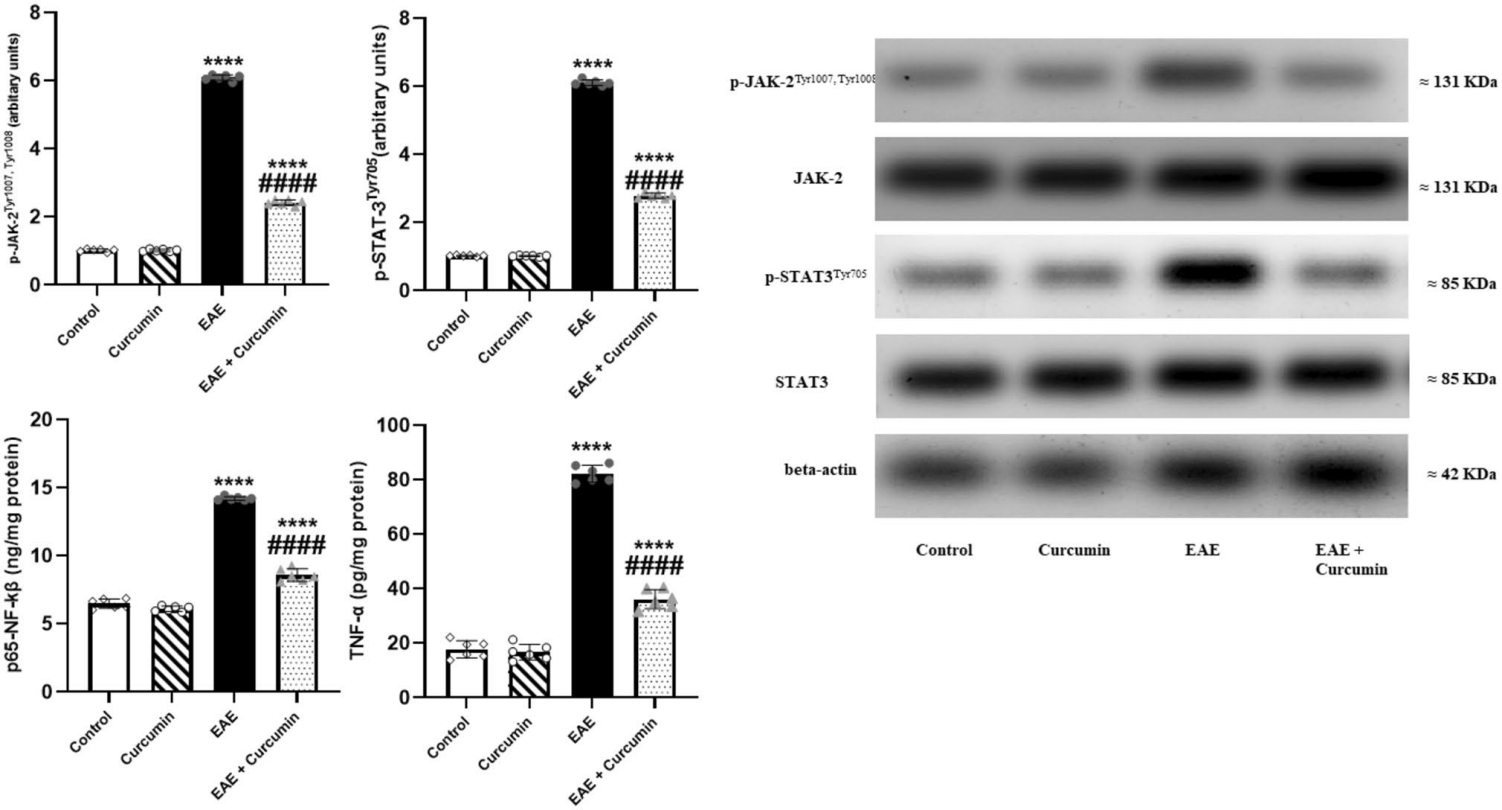
Curcumin altered EAE-induced neuroinflammation. Notably, p- JAK2^Tyr1007, Tyr1008^ and p-STAT3^Tyr705^ levels were determined using western blotting technique, while NF-kβ, and TNF-α levels were quantified using ELISA technique. Values are represented as the mean ± SD (*n* = 6/ group). **** significantly different from the control group at *p* < 0.0001, ^####^ significantly different from the EAE group at *p* < 0.0001, using one-way ANOVA followed by Tukey’s post hoc test

EAE neuroinflammation was manifested by a significant elevation in JAK2, STAT3, NF-kβ, and TNF-α levels (*F* (3, 20) = 9098.00, 10407.00, 863.00, and 554.20, respectively) by 5.99-, 5.96-, 2.19-, and 4.64-fold, respectively, compared to the control (*p* < 0.0001). Contrarily, curcumin suppressed these elevations and reduced these inflammatory markers levels by 60.20%, 54.32%, 39.53%, and 56.06%, respectively, compared to the EAE mice (*p* < 0.0001) (Fig. 12).

## Discussion

This study shed the light on the neuroprotective effect displayed by curcumin in the EAE model, which was illustrated by the amelioration of EAE-induced neuronal degeneration and demyelination, along with improvement in animals’ cognitive and motor functions. Although the curcumin neuroprotective effect has been reported in similar EAE models (Xie et al. 2009; Esmaeilzadeh et al. 2019; Mavaddatiyan et al. 2021; Manai et al. 2022; Sun et al. 2022), this study cleared, for the first time, the influence of curcumin on EAE-induced cognitive impairment. Furthermore, it demonstrated an unprecedented new molecular mechanism involved in this neuroprotective effect. This favorable effect is not only attributed to the inhibition of inflammatory cytokines, but also to the activation of the AMPK/SIRT1 pathway, which consequently activates the neurotrophic pathway CREB/BDNF, augments the antioxidant Nrf2, and suppresses the inflammatory pathway JAK2/STAT3.

Herein, the EAE mice model, which mimics MS, was created using SCH obtained from Sprague–Dawley rats, which acts as an antigen alternative to the myelin sheath due to their mutual structural similarity (Burrows et al. 2019). The SCH was emulsified in CFA, containing heat-killed *mycobacterium tuberculosis*, to provoke the immune system (Sanabria-Castro et al. 2020). Notably, the EAE model mimics MS in animals and represents a valuable tool for evaluating multiple drugs for managing MS (Burrows et al. 2019). In this context, mice that received EAE emulsion showed a great elevation in EAE clinical scores that denotes the ascending paralysis developed by EAE in mice, similar to prior studies (Berghmans et al. 2012; Mahfouz et al. 2017). Moreover, EAE mice showed a significant loss in appetite, reflected in their low total body weight, consistent with a previous study (Jelodar et al. 2021). On the other hand, treatment with curcumin effectively reversed the deleterious effect of EAE, as evidenced by the reduced EAE clinical scores and normalized total body, in accordance with other studies (Xie et al. 2009; Esmaeilzadeh et al. 2019).

EAE induction and cognitive impairment interplay together; hence, we verified this deterioration via NOR test and histological examination of the hippocampus. Herein, EAE induction significantly insulted mice’s cognitive function, as shown in the NOR test. Moreover, it triggered a remarkable inflammation in the hippocampus, as shown by H & E staining and, as previously noted (Kamarehei et al. 2019). In the same context, untreated neuroinflammation of the hippocampus provoked neurodegeneration and reduced the number of intact neurons, as demonstrated in the toluidine blue stain panels. Surprisingly, curcumin suppressed hippocampal inflammation and degeneration, as shown in the histological examination, justifying the protective influence of curcumin against EAE-induced cognitive impairments shown in the NOR test.

EAE deteriorated not only mice’s cognitive function, but also their motor function, muscle coordination, and grip strength as demonstrated by the open field, rotarod, and grip strength tests, respectively, consistent with earlier studies (van den Berg et al. 2016; El-Emam et al. 2021). Contrarily, treatment with curcumin minimized these deteriorations, agreeing with earlier studies (Xie et al. 2009; Esmaeilzadeh et al. 2019).

Recently, studies have strongly correlated the pathophysiology of MS with AMPK/SIRT1 signaling (Wang et al. 2016a, b, c; Ammar et al. 2022). AMPK is a serine/threonine kinase activated to amend metabolic stresses triggered by physiological or pathological stimuli (Ronnett et al. 2009). Neurodegenerative diseases provoke metabolic stresses; thus, AMPK activation has been highlighted as a promising neuroprotective target, where its upregulation could hinder neurodegeneration, oxidative stress, and neuroinflammation (Ronnett et al. 2009; Xu and Ash 2016). Similarly, SIRT1 is a nicotinamide adenine dinucleotide-dependent histone deacetylase, which imparts a fundamental role in neuroprotection through abolishing multiple factors involved in neuronal death in neurodegenerative diseases, such as oxidative stress and neuroinflammation (Manjula et al. 2020; Zhang et al. 2020). Interestingly, AMPK and SIRT1 mutually regulate each other, and their activation was proposed to have a vital role in neuroprotection (Ruderman et al. 2010). Several MS animal models showed downregulation of AMPK/SIRT1 signaling followed by prominent neurodegeneration (Wang et al. 2016c; Ammar et al. 2022). This was in line with our study where EAE mice showed a marked reduction in p-AMPK and SIRT1 levels. However, curcumin treatment boosts p-AMPK and SIRT1 levels, activating the neuroprotective axis, AMPK/SIRT1. Noteworthy, curcumin could activate AMPK either through direct binding, as previously shown using molecular docking, or by increasing cAMP levels (Liu et al. 2017; Iside, Scafuro et al. 2020).

The neuroprotective effect of AMPK/SIRT1 is based, to a certain degree, on the fact that the activation of AMPK/SIRT1 enhances neuronal survival by activating CREB, a transcription factor essential for the transcription of BDNF in neurons (Ng et al. 2015; Xu et al. 2018; D'Angelo et al. 2021). BDNF is a crucial neurotrophic factor that promotes neuronal survival, maintenance, and differentiation. Moreover, it facilitates the differentiation of the oligodendrocytes, the myelin-forming cells, and boosts the expression of MBP with subsequent enhancement of neuronal remyelination (VonDran et al. 2011; Fletcher et al. 2018). Our study showed the reduction of p-CREB, BDNF, and MBP levels in EAE mice which eventually led to neurodegeneration in the brain, supported by the decline in the LFB stains intensity in the corpus callosum (a highly myelinated part of the brain). Our results agree with Khodanovich et al., who reported a decline in the corpus callosum LFB staining intensity upon induction of MS in mice (Khodanovich et al. 2017). Contrarily, curcumin treatment efficiently modulated AMPK/SIRT1/CREB/BDNF signaling, supported by the incline in p-CREB and BDNF levels. Indeed, CREB/BDNF activation attenuates neuronal demyelination and death, as evidenced by the elevated MBP level in the whole brain, and increased LFB staining intensity in the corpus callosum. Notably, the increase of MBP expression by curcumin in EAE rats was previously reported by Mavaddatiyan et al. (2021). Interestingly, accumulating evidence indicates that BDNF is not only linked to neurogenesis but also has a vital role in cognitive function since BDNF enhances hippocampal synaptic efficacy (Lu et al. 2014; Miranda et al. 2019). Based on these findings, we can say that the preserved cognitive function observed in curcumin-treated mice is credited not only to the inhibition of hippocampal inflammation, as observed in the histological examination, but also to the boosting of BDNF levels.

Besides modulating BDNF and MBP, AMPK/SIRT1 signaling is crucial for Nrf2 activation (Shah et al. 2017). Nrf2 is a transcription factor that vitally suppresses oxidative stress and inflammation by modulating the transcription of vitagenes, such as SOD, glutathione, and sirtuins; thus, abating oxidative state and maintaining redox balance (Cornelius et al. 2013; Habtemariam 2019; Qi et al. 2020). Notably, oligodendrocytes are highly susceptible to oxidative stress owing to the high metabolic requirement associated with myelin sheath production, making oxidative stress a significant contributor to the pathogenesis of MS (Kim et al. 2020). In this context, the decline in the Nrf2 and SOD activities was noted in the EAE model and Nrf2 activators were highlighted as a promising agent for managing MS (Johnson et al. 2010; Lu et al. 2016). Herein, curcumin efficiently controlled oxidative stress by augmenting the activities of Nrf2 and SOD antioxidant enzymes, which were suppressed with EAE induction. This antioxidant activity of curcumin can be attributed to the activation of AMPK/SIRT1 signaling, which, in turn, activates Nrf2 which regulates SOD, resulting in the scavenging of excessive free radicals and attenuating of oxidative stress.

In addition to AMPK/SIRT1’s tremendous role in resolving neurodegeneration and oxidative stress, it effectively abates neuroinflammation (Xu and Ash 2016; Velagapudi et al. 2017). This was evidenced by its role in activating Nrf2, which suppresses the release of inflammatory cytokines (Yang et al. 2016; Ahmed et al. 2017). Moreover, AMPK/SIRT1 signaling inhibits the JAK2/STAT3 inflammatory pathway (Ni et al. 2014; Wojcik et al. 2018). The JAK/STAT pathway, specifically JAK2/STAT3, is involved in many physiological and pathological conditions and regulates the inflammatory response, cell proliferation, and immunity (Hou et al. 2018). Hyperactivity of the JAK/STAT pathway has been demonstrated in patients with MS, and indeed its inhibition might halt MS progression (Benveniste et al. 2014; Liu et al. 2014; Dang et al. 2021). It was noted that JAK activation leads to the phosphorylation of its downstream target, STAT, which, in turn, translocate inside the nucleus and binds to a promoter site. This results in the stimulation of NF-kβ signaling, which enhances the generation of inflammatory cytokines, mainly TNF-α (Harrison 2012, Elbaz, Senousy et al. 2018). These inflammatory cytokines enhance NF-kβ activation and stimulate the production of reactive oxygen species, further exacerbating inflammation and oxidative stress, resulting in a vicious cycle that eventually aggravates neuronal demyelination and death (Ramesh et al. 2012; Yan et al. 2018). Herein, EAE induction resulted in massive neuroinflammation besides oxidative stress, which was confirmed by high levels of p-JAK2, p-STAT3, p-NF-kβ, and TNF-α, and abundant inflammatory cell infiltrates in different brain regions in the histological examination, which was in harmony with a previous study (Liu et al. 2014). However, curcumin treatment arrested this induced inflammation, as shown by the biochemical and histological analysis, consistent with a prior study (Sun, Liu et al. 2022). Noteworthy, Sun et al. illustrated the ability of curcumin to amend JAK2/STAT3 in the spinal cord of EAE mice, by antagonizing Axl receptors (Sun, Liu et al. 2022). Interestingly, the inhibition of the JAK2/STAT3 pathway not only ameliorates NF-kβ signaling but also lessens the activity of microglia, amending neuroinflammation (Sun, Liu et al. 2022). Based on our findings, the anti-inflammatory effect of curcumin can also be attributed to the activation of the AMPK/SIRT1 pathway, as shown herein, along with curcumin’s ability to block the JAK receptor, inhibit STAT3 activation, and reduce STAT3 translocation into the nucleus, as previously mentioned (Farghadani and Naidu 2021).

## Conclusion

In conclusion, this study supports studies that demonstrated that curcumin had a neuroprotective effect in the EAE-induced MS in mice. Furthermore, it emphasized the ability of curcumin to amends not only EAE-induced physical disability but also EAE-induced cognitive impairments. Moreover, it showed that curcumin's beneficial action might be credited, at least in part, to its ability to activate AMPK/SIRT1 signaling. Indeed, AMPK/SIRT1 activation diminishes neurodegeneration, oxidative stress, and neuroinflammation. Additionally, curcumin is a natural drug with a wide safety margin, applied clinically in multiple diseases. This makes it a promising approach to attenuate neuronal demyelination and degeneration in MS, alleviating both cognitive and motor symptoms and improving the patients’ quality of life.

## Data Availability

The corresponding author can provide the data that were utilized to support this study’s conclusions upon reasonable request.

## Abbreviations

AMP: Adenosine monophosphate
AMPK: Adenosine monophosphate-activated protein kinase
ANOVA: Analysis of variance
BDNF: Brain-derived neurotrophic factor
CFA: Complete Freund’s adjuvant
CMC: Carboxymethyl cellulose
CNS: Central nervous system
CREB: Cyclic adenosine monophosphate response element-binding protein
EAE: Experimental autoimmune encephalomyelitis, or Experimental allergic encephalomyelitis
ELISA: Enzyme-linked immunosorbent assay
H&E: Hematoxylin and eosin
JAK/STAT: Janus kinase/signal transducers and activators of transcription
LFB: Luxol fast blue
MPB: Myelin basic protein
MS: Multiple sclerosis
NF-kβ: Nuclear factor-kappa beta
NOR: Novel object recognition
Nrf2: Nuclear factor erythroid 2–related factor 2
SIRT1: Silent mating type information regulation 2 homolog 1
SOD: Superoxide dismutase
TNF-α: Tumor necrosis factor-alpha
